# Microplastic Contamination from Ready-to-Cook Clams: Implications for Food Safety and Human Exposure

**DOI:** 10.3390/foods14223971

**Published:** 2025-11-19

**Authors:** Flavia Capuozzo, Angela Dambrosio, Salud Deudero, Michele De Rosa, Federica Ioanna, Nicoletta Cristiana Quaglia

**Affiliations:** 1 Department of Veterinary Medicine, University of Bari Aldo Moro, Str. P.le per Casamassima, km 3, 70010 Valenzano, Italy; flavia.capuozzo@uniba.it (F.C.); m.derosa1986@gmail.com (M.D.R.); nicolettacristiana.quaglia@uniba.it (N.C.Q.); 2 Instituto Español de Oceanografía, Centro Oceanográfico de Baleares, Muelle de Poniente s/n, 07015 Palma de Mallorca, Spain; salud.deudero@ieo.csic.es; 3 Competent Authority for Food from Animal Origin Inspection, Azienda Sanitaria Locale, 70124 Bari, Italy; fedeioanna@hotmail.com

**Keywords:** microplastics, ready-to-cook seafood, food safety, public health, risk assessment

## Abstract

Microplastic contamination in seafood has emerged as a significant concern for public health and food safety. Bivalve molluscs are especially vulnerable because of their filter-feeding behaviour, leading to the accumulation of different substances in seawater, including contaminants like microplastics. This study examines microplastic contamination by comparing commercially available ready-to-cook frozen and deep-frozen clams, assessing particle morphologies, dimensions, colours, and chemical identities. The Polymer Hazard Index (PHI) derived from the proportions of polymers in the samples and their hazard scores, whereas the Estimated Average Daily Intake (EADI) was determined based on per capita consumption and microplastic counts. The results indicated a significantly higher prevalence of microplastics in deep-frozen clams compared to frozen clams, with 2.58 ± 0.87 and 0.43 ± 0.13, respectively. EADI was estimated at 0.47 and 0.76 MP/kg(bw)/day for deep-frozen clams and frozen clams, respectively (before cooking). Our findings highlight the influence of industrial processing on microplastic contamination, other than the environmental contribution, with considerable implications for human exposure, underscoring the necessity for monitoring initiatives and regulatory policies to reduce microplastic exposure in seafood, thereby safeguarding food safety and public health.

## 1. Introduction

In recent years, microplastic pollution in aquatic ecosystems has become a critical global environmental and health concern. Microplastics, defined as plastic particles less than 5 mm, are ubiquitous in global aquatic environments and present a growing threat to food safety, especially for filter-feeding organisms such as bivalve molluscs [1,2]. These organisms can accumulate plastic particles, which may subsequently enter the human food chain. The growing acknowledgement of the potential toxicological effects of microplastics, including the release of chemical additives and the adsorption of environmental pollutants, has motivated wide research on their detection in food products, particularly in seafood [3,4].

Recent scientific literature has documented the presence of microplastics in different food sources, including bivalve molluscs, with findings varying according to environmental context and the specific organism studied [3,5].

Bivalve molluscs, including clams, mussels, and oysters, are of significant interest in microplastic research due to their ability to filter considerable quantities of seawater and accumulate minuscule particles. Multiple studies have examined the microplastic composition in fresh or frozen bivalve molluscs with shells, primarily identifying transparent fibres, in addition to lesser quantities of fragments and granules of environmental origin or resulting from plastic degradation [6,7,8,9,10,11]. Nevertheless, limited research has investigated contamination in processed bivalve molluscs, particularly shelled and ready-to-cook products, which are the forms most directly consumed by consumers. Examining processed bivalve molluscs is essential for assessing the actual risk to human health, as industrial processing methods like shelling, washing, freezing, and packaging may introduce supplementary microplastics or modify the size and distribution of pre-existing particles [9]. Clams are primarily marketed fresh, although they are also available as frozen or deep-frozen products, either whole or shelled. The main difference between freezing and deep-freezing is the speed at which the product freezes. In deep-freezing, the rapid decrease in temperature promotes the formation of small ice microcrystals, which helps to preserve the intrinsic texture, organoleptic properties, and overall biochemical integrity of the food. In contrast, standard freezing proceeds more slowly, resulting in the development of larger ice crystals that can damage cellular structures and lead to greater qualitative deterioration compared with deep-freezing [12].

This study aims at quantifying and characterising the presence of microplastics in ready-to-cook frozen clams with shells (FC) and ready-to-cook deep-frozen clams without shells (DFC) and at assessing the impact of industrial processing on microplastic contamination. This experiment design provides current and relevant data for food safety risk assessment, as well as the implementation of targeted policymaking strategies aimed at reducing contamination in products intended for human consumption. In particular, this study is one of the few scientific contributions that focuses on processed bivalve molluscs, filling a gap in the literature and providing crucial information for health authorities, producers, and consumers.

## 2. Materials and Methods

### 2.1. Sampling

**Figure 1:**
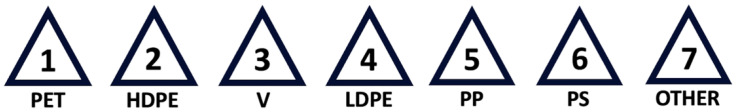
Resin Identification Codes (RIC) according to Advancing Standards Transforming Markets (ASTM) D7611/D7611M-2 (2022) [13].

Five samples of vacuum-packed ready-to-cook frozen clams with shells (FC) and five samples of deep-frozen clams without shells (DFC) packaged in plastic bags with preserving liquid were purchased from two retail markets and three GDOs in Bari (Apulia, Italy). All samples were stored at −18 °C until analysis. Six out of ten sample packages (all frozen clam and one deep-frozen clam samples) were made of plastic coded 7 that refers to all non-recyclable plastic composites not included in the categories from 1 to 6 in the Resin Identification Codes—RIC according to ASTM D7611/D7611M-21 [13] (1 Polyethylene Terephthalate, 2 High-Density Polyethylene, 3 Polyvinyl Chloride, 4 Low-Density Polyethylene, 5 Polypropylene, 6 Polystyrene). Whereas the other 4 sample packages (all deep-frozen samples) were made of low-density polyethylene (LDPE), coded 4 in RIC (Figure 1).

The vacuum-packed frozen clams were collected with dredges from the Black Sea (FAO area 37.4.2) and from the central-western Pacific Ocean (FAO area 71) and belonged to the species *Chamelea gallina* and *Paphia textile*, respectively, as reported on the labels. The deep-frozen clams without shells kept in preserving liquid were collected with dredges in the Black Sea, the north-western and central-western Pacific Ocean (FAO areas 37.4, 61 and 71, respectively) and belonged to the species *Meretrix lyrata*, *Paphia textile* and *Chamelea gallina*, as indicated on the labels. All samples were frozen between the end of 2023 and the beginning of 2024, as reported on the labels.

### 2.2. Extraction and Detection of Microplastics

Microplastics were extracted employing oxidative digestion followed by density separation, according to Li et al. (2016) [7].

Each sample was divided into three replicates to ensure accuracy, and aliquots of 10 g were digested at 60 °C for 24 h in 30% hydrogen peroxide (H_2_O_2_) (1:20 *w/v*) in an oscillating incubator (ASAL s.r.l. Cernusco, Milano, Italy) at 80 rpm, followed by an additional 24 h at room temperature without oscillation. For deep-frozen clam samples, each aliquot included both clam tissues and preserving liquids. Density separation was performed by adding NaCl to distilled water (1.2 g mL^−1^); the solution was incubated overnight and then filtered before being added to the sample digestates (≈800 mL for each replicate) in 1 L cylinders; then they were rapidly covered with aluminium foil and maintained at room temperature overnight. The supernatants were then filtered with a metal vacuum pump system (KNF Flodos AG, Sursee, Switzerland) through nitrocellulose filter membranes (1.2 μm pore size and 47 mm diameter) (Axiva Sichem Biotech, Delhi, India). Each filter was subsequently transferred in covered glass Petri dishes (Fischer Scientfic, Segrate, Milano, Italy) to allow the drying at room temperature.

Procedural blanks were performed with the chemical solution alone to assess microplastic airborne contamination. Sample particles similar to blank particles were excluded from results.

After drying, each sample and procedural blank filter was observed under a stereomicroscope (Nikon, Moncalieri, Torino, Italy) to detect and count potential MPs. Particles were classified by morphologies (fibres, fragments, films, granules), dimension categories (5–500 µm, 501–1000 µm, 1001–5000 µm, >5000 µm), and colour following the guidelines by Hidalgo-Ruz et al. (2012) [14]. Images were acquired with a digital camera (Nikon X_Entry, Tokyo, Japan).

### 2.3. Polymer Identification of Microplastics

Particle chemical identification was performed employing a Nicolet™ iS50R FT-IR Advanced KBr Gold Spectrometer (Thermo Fisher Scientific™, Segrate, Milano, Italy) with built-in attenuated total reflectance (ATR) (High Energy Transmission Single Reflection AR-coated Diamond Crystal), and spectra were acquired in the range of 400 to 4000 cm^−1^. Each FT-IR spectrum included 16 scans, measured within the wavelength range of 400 to 4000 cm^−1^ with a resolution of 4 cm^−1^. The selected particles were first detected under the stereomicroscope and then analysed on the filter surface with FT-IR.

Only spectra with a correlation score ≥ 0.70 between sample polymers and reference polymers from the spectral database (Thermo Fisher Scientific™) were considered valid and included in the results, ensuring a robust identification approach.

During the FT-IR ATR analyses, in order to minimise the risk of laboratory contamination, procedural blanks were systematically performed when samples were manipulated by exposing glass Petri dishes with empty filters to the laboratory environment. No polymers were identified in the blanks, confirming that the spectra obtained from clam samples were not biassed by laboratory contamination.

### 2.4. Quality Assurance/Quality Control Measures

To minimise contamination, strict precautions were implemented during the entire experiment.

All staff members wore a white 100% cotton lab coat and nitrile gloves all the time; the laboratory door remained closed, and movements in and out of the room were kept as minimal as possible. All laboratory equipment was made of glass or metal and rinsed three times with filtered distilled water before and after use and covered with aluminium foil to prevent airborne MP contamination. Work surfaces were kept clean using 100% cotton towels soaked in 90% filtered ethanol.

All fluids employed during the experiment (distilled water, saline solution, and hydrogen peroxide) were filtered before use with membrane filters with a pore size of 0.45 μm and a diameter of 47 mm (Axiva Sichem Biotech, Delhi, India) and kept in pre-rinsed glass flasks covered with aluminium foil.

### 2.5. Statistical Analysis

Microsoft Excel and RStudio software (RStudio 2025.05.1+513) (macOS, Sequoia 15.3.1, 24D70) were used to analyse the data, with a significance level of α = 0.05. Statistical analyses were conducted in order to find both descriptive patterns and inferential differences.

Descriptive statistics (mean, median, and standard deviation) were calculated both to provide a general overview of the data and to select appropriate inferential tests.

A boxplot graph was applied to evaluate data distribution, defining baseline contamination levels and differences between the frozen clams with shell (FC) and deep-frozen clams without shell (DFC) samples. Due to the non-normal distribution, non-parametric tests were performed. Multiple linear regression, complemented by the negative binomial model, was employed to further understand the distribution of data, taking into account morphotype and size classes, in order to assess how variables influenced contamination levels.

The Mann–Whitney U test was used to determine overall differences between the samples and median values. When significant results were obtained, post hoc comparisons were carried out using pairwise Wilcoxon rank-sum tests with Bonferroni, Holm, and Hochberg corrections to ensure transparency in inferences about pairwise group differences.

The Pearson’s chi-square test was applied to detect associations between microplastic categories like morphology, colours, and dimensions, highlighting whether certain categories (e.g., transparent fibres) were represented more in specific sample groups than others, whereas Cramér’s V index defined the strength of association between the microplastic categories (morphology, dimensions and colours). Finally, the chi-square test with Monte Carlo simulation assessed whether there were differences in polymer types between the group samples.

### 2.6. Polymer Hazard Index (PHI)

To assess the risk for public health through the consumption of ready-to-cook frozen clams and deshelled deep-frozen clams contaminated with MPs, the chemical toxicity of various polymer types is taken into account [15]. Both the concentration and the chemical identities of MPs have been taken into account in order to assess the possible hazards of MPs in the samples of this study [16]. Using the following formula, the polymer hazard assessment of MPs was determined:

(1) $$PHI=\sum P_{n}*S_{n}$$

where *PHI* is the calculated polymer hazard index caused by MPs, *P_n_* is the percent of specific polymer types identified in the group samples, and *S_n_* is the hazard scores of polymer types of MPs derived from Lithner et al. (2011) [16].

### 2.7. Estimated Average Daily Intake (EADI)

Risk assessment for consumer health’s exposure to MP assumption through ready-to-cook frozen clams and deshelled deep-frozen clams was performed by calculating the Estimated Average Daily Intake (EADI).

The EADI was calculated based on average microplastic content (MP/g) in each group sample type and average per capita annual consumption of 30 kg of processed bivalves [17], employing the formula used by Li et al. (2022) [18]:

(2) $$EADI=\frac{C_{MP}\times IR}{BW}$$

where *C_MP_* = mean count of MP/g of tissue; *IR* = annual per capita ingestion rate; *BW* = mean body weight (~70 kg in food risk research). The *IR* was obtained from consumption data from Istituto Nazionale di Statistica [17]. Annual *IR* values (g/year) were converted into daily averages by dividing by 365.

This type of food is consumed after cooking, in particular by frying. Li et al. (2022) [18] observed significant differences between different methods of cooking clams, assessing that different cooking methods increased or decreased the relative abundance of fibres, fragments and granules. Since the food investigated in this study is mainly consumed after frying, and the authors observed an increase in fibres of 75% and a decrease in fragments and granules of 15% and 10%, respectively, the EADI post-cooking was calculated taking into account these percentages.

## 3. Results

Analysis of frozen clams with shell (FC) and deep-frozen clams without shell (DFC) revealed significant differences in microplastic contamination.

Procedural airborne controls confirmed minimal contamination, with DFC controls averaging 21.2 ± 13.81 MPs (0.11 ± 0.07 MP/mL) and FC controls averaging 20 ± 2.45 MPs (0.1 ± 0.01 MP/mL), indicating that contamination originated from bivalve tissues rather than laboratory artefacts.

**Figure 2:**
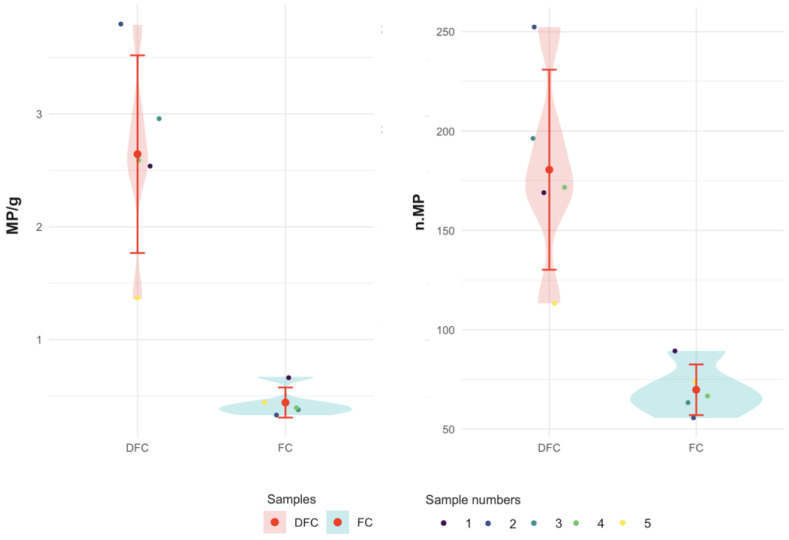
Microplastic distributions across samples DFC (from 1 to 5) and FC (from 1 to 5) with mean values and SD.

Descriptive statistics revealed that DFC samples contained a maximum mean of 252.33 ± 142.78 MPs (DFC 2), whereas FC samples had a maximum mean of 89.33 ± 60.28 MPs (FC 1). DFC samples exhibited greater variability, as shown by higher standard deviations (SD 152.47 in the sample DFC 1 compared to 60.28 in FC 1) and a wide range of values (Figure 2).

**Figure 3:**
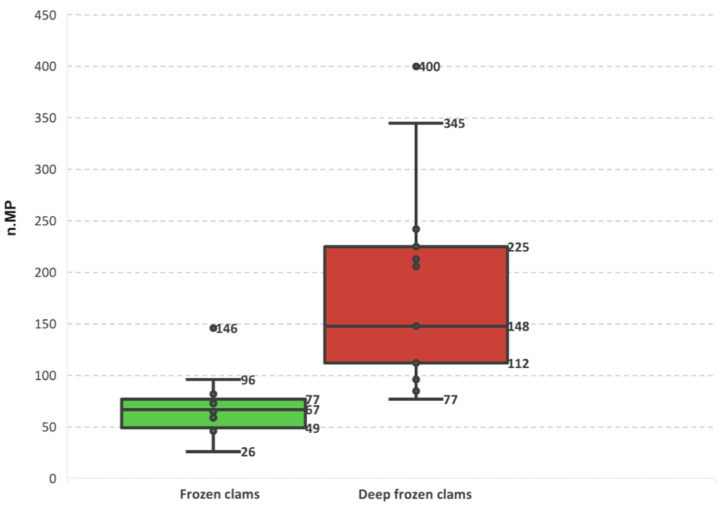
Distribution of microplastic counts recovered from FC and DFC and across each sample.

Wider interquartile ranges (IQR—67 and 148 for the FC and DFC, respectively) and more pronounced outliers (400 particles in replicate b, DFC 2 sample) confirmed that the variability between DFC replicates was considerably greater. In contrast, FC sample replicates showed lower counts and more homogeneous distributions (Figure 3).

Regression analysis indicated that replicate effects were not statistically significant, thereby validating measurement consistency. The negative binomial model further confirmed the high variability of DFC samples caused by outliers, reflecting the heterogeneous nature of microplastic contamination.

**Figure 4:**
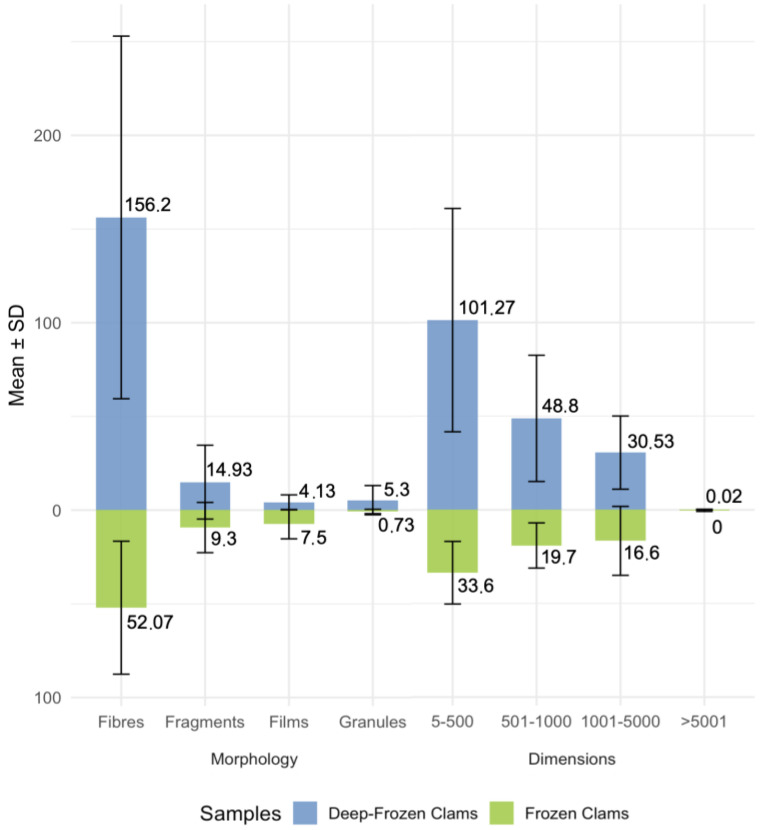
Mean values and SD of plastic particle morphologies and dimensions of FC and DFC.

Analysing the morphology and size distribution, most particles were fibres and fell within the 5–500 µm range of dimensions (Figure 4).

**Figure 5:**
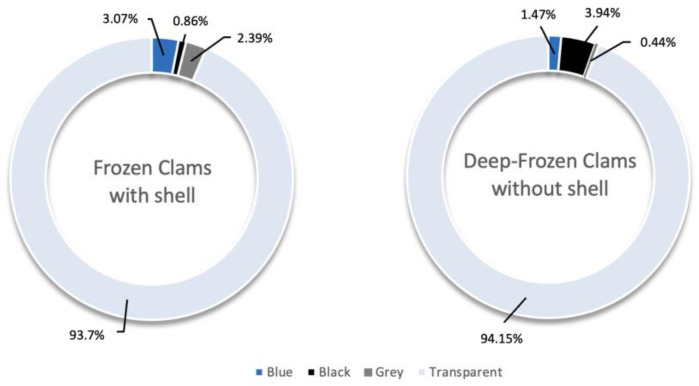
Colours of MPs in FC and DFC samples, expressed in %.

Transparent was the dominant colour, although DFC also showed fragments and black-coloured particles and FC blue-coloured particles (Figure 5).

The Mann–Whitney U test corroborated the significant differences in microplastic contamination levels between the FC and DFC samples.

However, in the Wilcoxon rank-sum test, the post hoc pairwise comparisons (45 tests), no pair was significant after corrections of Bonferroni, Holm, and Hochberg. The significant result of the Mann–Whitney U test indicates that there is an overall difference between FC and DFC, but it does not clearly emerge as a significant difference between the samples of the two types of clams.

The Pearson’s chi-square test and Cramér’s V index results indicated that the proportions of fibres, fragments, films, and granules are statistically significant with a moderate association (V = 0.21) between the two types of samples. The particle size classes highlighted a moderate association, whereas the colour composition showed an extremely strong difference between FC and DFC (*p* < 0.001; V = 0.93), with the particle colours strongly correlated with the sample types.

**Figure 6:**
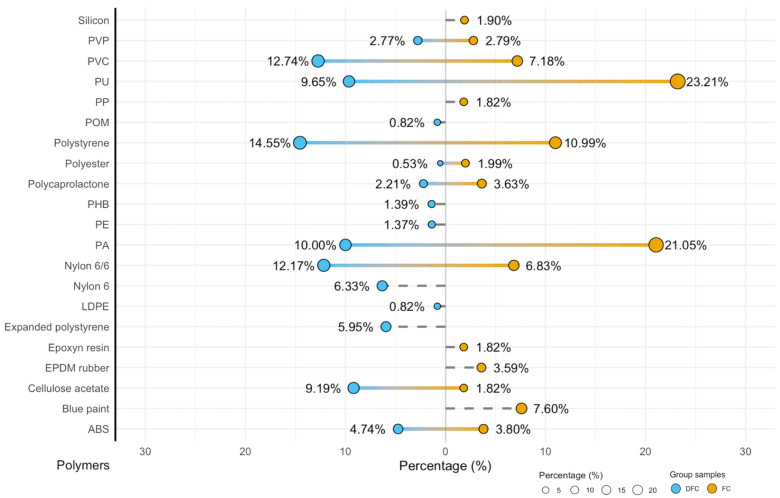
Polymers detected with ATR FT-IR.

The FT-IR ATR analysis of ready-to-cook FC and DFC revealed a heterogeneous profile of microplastic contamination and a statistically significant difference between both type samples (chi-square test with Monte Carlo simulation). A wide variety of polymers was identified, including polyamides (polyamide—PA, Nylon 6 and Nylon 6/6), polystyrene (PS), polyurethane (PU), polyvinyl chloride (PVC), acrylonitrile butadiene styrene (ABS), polyethylene (PE), polyester, cellulose acetate, polymer composites such as epoxy resins, paints, ethylene-propylene diene monomer (EPDM) rubber, and silicone. Polymer proportion distributions are shown in Figure 6.

The difference in polymer identities has important implications for human health. Clams with shells exhibited higher proportions of PU (23.21%) and PVC (7.18%), both classified as high-hazard polymers (level 5) [16], resulting in a total Polymer Hazard Index of 439,867.82. In contrast, de-shelled clams contained relatively higher percentages of PS (14.55%) and Nylon 6/6 (12.17%), with a slightly lower total hazard index (303,485.84). Other polymers, including PCL, EPDM rubber, PVP, and cellulose acetate, lacked hazard data and were thus defined as not classified (N.c.) (Table 1).

The Estimated Average Daily Intake (EADI) of MPs via consumption of ready-to-cook clams was also calculated based on the average microplastic content per gram of tissue (MP/g) detected in each sample type and the average annual per capita consumption of 30 kg of processed bivalves in Italy [17] with an average body weight of 70 kg. The EADI was determined both before and after cooking since these types of food are consumed prior to cooking (as defined by the category ready-to-cook), in particular after being fried.

**Figure 7:**
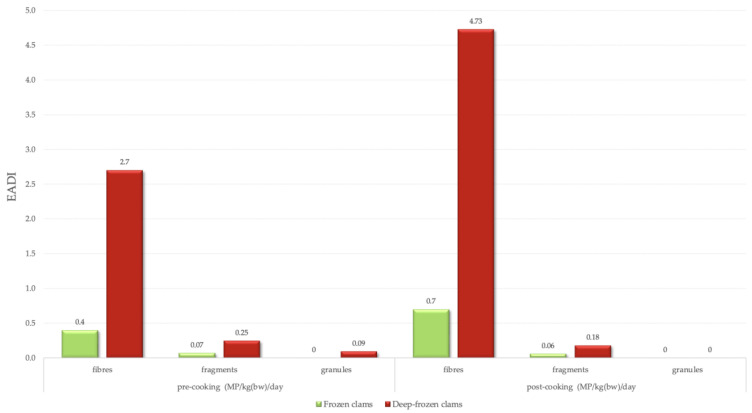
Microplastics Estimated Daily Intake (EADI) per day per kg of average body weight (~70 kg).

Results indicate that EADI for fibres increased in both group samples, reaching 4.73 and 0.7 MP/kg(bw)/day for DFC and FC, respectively. Whereas for other morphologies, frying decreased the counts but showed a lower impact (Figure 7).

## 4. Discussion

The evaluation of human exposure to microplastics (MPs) through bivalve consumption is gaining increasing attention in food safety and environmental health, particularly in Mediterranean countries where shellfish are a key dietary element. This study examines ready-to-cook frozen with shell (FC) and deep-frozen deshelled clams (DFC), which are products commonly consumed in Mediterranean countries, including Italy. The processing flow for these two products is different and may influence differently the contamination from microplastics of this product. Understanding these differences is crucial for assessing consumer exposure, developing efficient monitoring strategies, and making policy decisions to reduce microplastic intake from seafood.

### 4.1. Microplastic Particle Distributions Across Sample Groups DFC and FC

The findings of this study offer strong evidence of considerable microplastic contamination in the analysed samples.

A clear difference in microplastic contamination was observed between FC and DFC. FC samples showed contamination levels, with total counts ranging from 55.67 ± 16.74 to 89.33 ± 60.28 items, whereas in DFC samples were considerably higher, ranging from 113.33 ± 1.53 to 252.33 ± 142.78 items (Table 2).

These findings are consistent with the assumption that the shell of frozen clam samples provided a physical barrier, limiting direct contact between soft tissues and external contaminants. According to this theory, Zhu et al. (2022) [19] showed that undamaged shells reduced secondary contamination at later stages of the production chain. Furthermore, frozen clams with shells were vacuum-packed with plastic bags coded 7 by the Resin Identification Codes (RIC), a category including polycarbonate and multilayered plastics that release microparticles or chemical additives under certain conditions such as temperature, storage conditions, fat content of specific food and contact time of packaging with food [20]. Thus, although vacuum packaging has been intended to extend shelf life, it may increase microplastic contamination through handling or particle loss.

In contrast, the higher microplastic counts in DFC may be related to both the absence of this natural barrier and the influence of industrial packaging and storage conditions [21]. Regression results confirmed a significant effect of the type of processing technologies on the number of microplastics isolated. In fact, whereas the intra-sample replicate influence of DFC was minimal, taking into account the inter-sample replicates, dispersion remained critical. This was caused by few extreme values reflecting the unpredictable nature of microplastic contamination, which may originate from heterogeneous environmental sources or processing variability [22].

Prolonged contact with packaging materials, combined with liquid media (i.e., the preserving liquid), may result in an increase in microplastics in edible tissues, according to previous studies that demonstrated microplastic fragmentation into smaller particles or the release of chemical additives in food under certain environmental conditions, such as mechanical stress, temperature fluctuations, and the presence of liquids [23,24]. According to Chinglenthoiba et al. (2025) [25], the preservation liquid itself may act as a medium for retaining or concentrating microplastics, thereby influencing the count.

The difference between the two types of samples was not confirmed by the pairwise comparison tests. This could highlight the difficulties of studying microplastic contamination in food matrices, where biological variability and complex processing flows interact to produce heterogeneous results [26].

Some studies found that processed bivalves showed a higher number of MPs compared to fresh products due to packaging, post-harvest handling, processing and storage methods [24,25]. In contrast, Avio et al. (2015) [27] showed that in controlled laboratory conditions, the accumulation of microplastics in mussels was primarily due to environmental contamination, suggesting that industrial processes alone cannot entirely explain the MP contamination. In light of the results of this study, lower counts were reported by Nalbone et al. (2024) [28] with 0.19 ± 0.21 MP/g in processed clams (in brine and vacuum frozen). Hence, different species, processing, preservation techniques and MP environmental pollution made a significant impact on microplastic content in seafood [29].

Industrial equipment, such as conveyor belts subjected to mechanical wear and friction, washing systems, especially those using plastic components or subjected to wear, and polymeric packaging, degrading over time, are all potential sources of microplastics in food processing environments [25,30]. These sources highlight the need to strengthen preventive strategies with the hazard analysis and critical control point (HACCP) in the seafood processing chain, such as integrating filtration technologies, air quality control, minimising deshelling, controlling packaging environments and optimising packaging materials, in order to safeguard public health [21,23,24,25,31]. These possibilities highlight the need to conduct interdisciplinary research that links food technology, environmental science, and food safety to face the challenge of risk assessment related to microplastic exposure in human diets [3,32].

### 4.2. Physical Characteristics of Isolated Particles

Both group samples were dominated by fibres and smaller size classes (5–500 μm) (Figure 4). Fibres reached a mean of 156.2 in DFC, suggesting a strong link with textile-derived contamination and secondary fragmentation processes through abrasion, wastewater effluents, degradation of synthetic materials and surface–material interactions [33]. This observation is consistent with previous studies indicating that fibres constitute one of the most prevalent morphotypes in aquatic and food matrices [34,35]. The smaller size category is especially worrying because of enhanced bioavailability and the capability of crossing biological barriers, like epithelial barriers in humans, thus accumulating in tissues and causing oxidative stress and inflammatory responses [22,32,36,37,38]. Despite these concerns, there is still a lack of a quantitative dose–response relationship that would allow reliable risk assessments [39].

Colour analysis provided further information. Transparent, black and blue microplastics were predominating, reflecting their widespread use in packaging and industrial applications. The recurrent presence of transparent items is especially concerning for food safety, as these particles can remain visually undetected in food matrices, complicating monitoring and risk assessment [3]. Black and blue colours in DFC samples showed a significant occurrence, most likely related to potential contamination during technological processes involving inevitable contact with synthetic materials [40].

These findings highlight the difficulty in capturing the complexities of microplastic contamination, as well as the importance of incorporating microplastic monitoring into industrial processes that are directly linked to the food supply chain [31]. Preventive measures, such as the implementation of filtration technologies, improved wastewater management, and the redesign of synthetic materials, may reduce contamination levels. Addressing microplastic contamination is not only an environmental issue but also a critical concern for public health. Indeed, prolonged and minimal exposure to MPs may act as a vector for chemical contaminants and microbial biofilms, increasing the toxicologic and microbiological risk to human health [31].

Nonetheless, epidemiological studies on humans are limited, leaving long-term health effects uncertain [41,42]. Future research should focus on developing standardised protocols for sampling, detection, and quantification, as well as establishing maximum allowable levels based on particle type, size, and polymer identity. Otherwise, microplastic pollution threatens to become a permanent aspect of the food system, impacting both environmental sustainability and human health safety [21,37].

### 4.3. Chemical Identities of Isolated Particles

The FT-IR ATR analysis revealed a heterogeneous polymeric profile in both FC and DFC samples.

Polyamides, such as Nylon 6/6 and Nylon 6, were frequently detected in both samples. This is probably due to the wide use of these polyamides in aquaculture nets, fishing gear, and handling equipment. This indicates that the marine environment is the main source of contamination [43]. Ding et al. (2021) [4] emphasised how widespread synthetic fibres are in bivalve molluscs worldwide. These results demonstrate that textile fibres, fishing nets, and aquaculture ropes all significantly contribute to the contamination of bivalve MP.

Polystyrene (PS and EPS) emerged as the most prevalent polymer, particularly in DFC samples, indicating a strong influence from packaging materials, specifically EPS boxes commonly used in seafood transportation and storage. Previous studies revealed similar findings, with PS frequently associated with food packaging and EPS containers used in seafood distribution (Table 3).

Experimental studies further indicate that bivalves are capable of ingesting PS microspheres, which can accumulate in edible tissues [34].

PVC and PU, detected in different samples, are commonly associated with tubing, insulation materials, machinery components, consumer packaging, industrial resins, and synthetic fibres, implying that contamination of the analysed bivalves comes from both environmental sources associated with aquaculture facilities and post-harvest routes. Comparable mixed polymer profiles have been documented in European retail bivalves, with clams and mussels typically containing PS, PE, PP, and polyamides. This study found higher levels of PU and PVC than previously reported, possibly due to packaging or processing environments [46,47].

Paints and epoxy resins were found in the majority of FC samples, indicating that contamination could have come from boat coatings, aquaculture facilities, or direct shell contact with painted surfaces. This pattern is consistent with previous findings indicating that local anthropogenic sources influence polymeric profiles in bivalves [48].

In contrast, DFC samples exhibited a greater prevalence of biopolymers and bio-based plastics, including cellulose acetate (up to 19.23%) and PHB (up to 3.13%). These results may indicate the employing of innovative packaging materials promoted as biodegradable and contemporary industrial additives during processing. According to Abbasi et al. (2025) [49], cellulose acetate is one of the most commonly identified microfibres in marine ecosystems, often associated with cigarette butts, packaging materials, or filtration systems. PHB, which is frequently marketed as a safer alternative to traditional food packaging due to its accelerated decomposition, may produce degradation derivatives with unclear toxicological profiles [50,51,52].

PVP, a soluble polymer commonly used as an additive in food and pharmaceuticals, was found in many samples. Its presence may indicate additive residues or contamination during industrial processing [53].

PS, PVC, PU, and various polyamides were found in significant quantities. In contrast, PP and PE, typically prevalent in marine bivalves, were found in lower percentages, potentially indicating both environmental variability and packaging contamination.

Furthermore, FC samples contained blue paints, epoxy resins, and EPDM rubber that were linked to heavy metals and other hazardous additives, primarily originating from environmental sources, such as external surfaces, coatings, or interactions with industrial and maritime facilities, whereas DFC samples (EPS, cellulose acetate, and PHB) were primarily derived from industrial and packaging sources [54].

The observed differences in polymer distribution between the two groups could be attributed to many variables in the food production process, such as variations in processing techniques, storage conditions, and handling practices. These factors highlight the importance of rigorous quality control protocols throughout the food production process to ensure consistency and safety [30,55].

The vacuum-sealed bags were made of a plastic classified as 7 according to the Resin Identification Codes [13], which represents a heterogeneous category of materials with different chemical-physical properties and the potential for migration or particle release. This makes contamination from packaging concrete, occurring through both chemical transfer (migration of additives or monomers) and physical contamination (abrasion or release of microfragments due to handling, compression, or cutting of the film) [56,57]. This emphasises the importance of targeting risk management strategies for both marine pollution and food-contact packaging and processing environments. In this context, the adoption of alternative non-plastic barriers may reduce the risk of post-harvest contamination [58].

The identification of packaging-associated polymers raises concerns regarding food safety and public health. Bivalves are recognised as a major dietary source of micro- and nanoplastics [3]. They filter seawater that may contain particles and fibres derived from the degradation of marine plastic waste as well as urban or industrial effluents, indicating the level of pollution in the contiguous marine ecosystem and identifying the primary contaminants. Post-harvest contamination, also known as secondary contamination, includes packaging, handling, freezing, and any other processes that introduce or redistribute plastic particles and compounds [59]. According to other researchers, variables like temperature, contact time, compression (such as vacuum), and material type (polycarbonate, PVC, or multilayer laminates) influence both the mechanical detachment of microparticles and the migration of chemicals (like phthalates and bisphenols) [60]. Therefore, in our study it is reasonable to assume that some of the detected polymers come from packaging sources, considering the presence of both code-7 packaging and vacuum packaging processes. Products should better be packaged in glass since it provides practical, non-plastic barriers; if not possible, it is recommended to use materials that come into contact with food with a known chemical profile and are easier to recycle. These steps can reduce the migration of hazardous chemical compounds as well as the physical release of microfragments [61].

Different polymer types may influence the nutritional quality, texture, and overall safety of food products [62]. Certain polymers may be more vulnerable to degradation or contamination, posing potential health risks to consumers [63]. The European Food Safety Authority [64] has emphasised the potential for plastics to transport additives such as bisphenols or phthalates, as well as hydrophobic environmental pollutants, despite the incomplete characterisation of their toxicological effects on humans. Current evidence suggests that the total amount of microplastics consumed through seafood is relatively low in comparison to other dietary sources; however, the long-term exposure to various polymers and additives raises serious concerns [2]. The relationship between the presence of plastic particles and the established toxicological risk to humans is complex and depends on a variety of factors such as fragment size, polymer type, associated additives, and overall exposure level. The identification of polymers linked to packaging coded 7 reinforces the necessity for risk management strategies that prioritise the supply chain and packaging, rather than exclusively emphasising consumer education [3]. Populations at risk, such as high seafood consumers and those living near the coast, may be more vulnerable.

Consequently, these findings highlight the importance of incorporating microplastic monitoring into food safety assessments in order to establish regulatory limits for acceptable contamination levels.

### 4.4. Polymers Hazard Index (PHI) and Estimated Average Daily Intake (EADI)

The analysis of plastic polymer contamination in frozen clams, both with and without shells, reveals significant differences in microplastic composition and associated chemical hazards between the two sample groups.

The Polymer Hazard Index (PHI), which is calculated as the sum of hazard scores per polymer for each sample group, reveals significant chemical risks associated with the detected microplastics. In FC samples, the total PHI was estimated at 439,867, while in DFC samples, the total PHI was estimated at 303,485.

Siddique et al., (2025) [65] documented significantly lower PHI values between 565.40 and 659.26 in marine fish from the Bay of Bengal, categorising them as “Danger” risk. Likewise, Simon-Sánchez et al. (2024) [66] detected significant polymer-associated hazard levels in sediments from the Limfjord, Denmark, although these were not quantified as a total PHI value. The comparisons reveal that the PHI levels identified in the current FC and DFC samples are significantly higher than those documented in aquatic or sediment environments, highlighting a considerably greater chemical hazard potential in food-related matrices.

Notably, PU, PVC, and ABS exhibited the highest hazard levels (5), according to the classification proposed by Lithner et al. (2011) [16], indicating severe environmental and human health risks. Polymers with moderate hazard levels (3–4) included PA, Nylon 6/6, and polyester, whereas polymers such as polystyrene and polyethylene demonstrated lower hazard levels (1–2).

The prevalence of high-hazard polymers in both sample types raises serious concerns about the potential transfer to humans of toxic substances, including endocrine-disrupting additives, through seafood consumption. PU, PVC, and ABS contain hazardous additives and monomers, such as phthalates, bisphenols, and aromatic amines, linked to endocrine disruption, neurotoxicity, and carcinogenicity in experimental models [16].

The observed differences between clams with and without shells could be attributed to post-harvest handling, such as freezing and depuration, which may affect polymer retention, as well as biological filtration processes [67]. The presence of polystyrene and nylon in de-shelled clams may indicate contamination from production or processing environments or packaging materials, whereas elevated levels of PU and PVC in shelled clams may suggest a higher retention of high-molecular-weight, persistent polymers from the marine environment [68].

Estimating the average daily intake is fundamental to assessing the risk associated with the consumption of these types of food contaminated with MPs. The Estimated Average Daily Intake (EADI) is defined as the recommended level of nutrient consumption based on observed or experimentally determined estimates of intake by healthy individuals, assumed to be adequate for maintaining health [69]. In this study, the EADI of MPs was calculated based on the average abundance of particles detected in FC and DFC, in combination with per capita annual seafood ingestion rate data, estimated at approximately 30 kg per year for processed bivalves [17].

**Figure 8:**
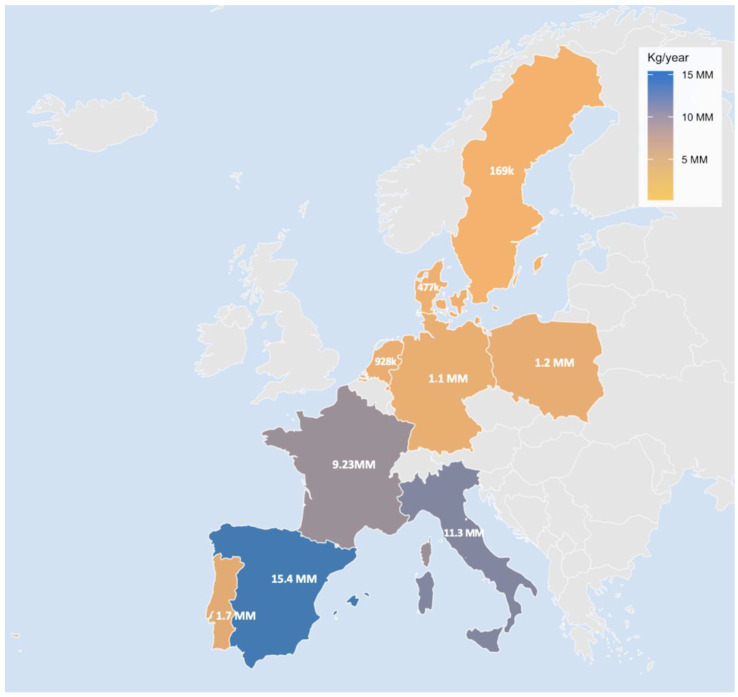
Annual consumption of processed bivalve molluscs (dried, frozen, salted or in brine) per region according to EUMOFA (2023) [69].

Particular attention should be paid to the specific context of Southern Europe. Clams, mussels, and other bivalves play an important role in the region’s culinary and cultural traditions. According to EUMOFA (2023) [70], consumption of processed bivalves (dried, frozen, salted or in brine) in Southern Europe is substantially higher than the European average, positioning these populations at greater potential risk of chronic dietary exposure to MPs (Figure 8).

Recent experimental findings show that traditional cooking methods (boiling, steaming, and frying) can influence the detectable concentrations of microplastics based on their morphology [18]. Including these correction factors in the calculation leads to an estimated daily intake from 5.07 to 4.91 MP/kg(bw)/day for DFC consumers and from 0.48 to 0.76 MP/kg(bw)/day for FC consumers.

These values indicate that DFC represents a substantially greater source of microplastic exposure than FC, consistent in both pre- and post-cooking assessments. In contrast, FC has lower MP exposure; however, there is a relative increase following cooking due to the elevated fibre content in FC samples prior to cooking and a 75% increase post-cooking (i.e., frying), while fragments and granules endure a reduction [18].

Based on these findings we can suppose that food processing and packaging appear to play a critical role in determining microplastic intake, in some cases increasing the MP content of the raw product. These observations highlight the need to consider post-harvest handling, processing and culinary practices when assessing dietary microplastic exposure.

This regional pattern highlights a prevalent exposure pathway in bivalve consumer populations. Previous studies demonstrated that the South of Italy represents a critical case due to the convergence of cultural consumption patterns and elevated per capita intake, which increases the risk of chronic exposure to MPs [10]. The potential health implications of microplastic ingestion, such as intestinal barrier disruption, inflammatory responses, and systemic distribution, raise concerns about long-term gastrointestinal, metabolic, and immunological health outcomes, necessitating targeted monitoring, public awareness, and risk reduction strategies [71].

The combination of PHI and EADI data indicates that consumers of these seafood products may be exposed to toxicologically significant levels of hazardous substances. These polymers may include endocrine-disrupting additives, bisphenols, phthalates, and other hazardous monomers, leading to chronic health risks from repeated dietary exposure, such as endocrine, neurodevelopmental, and carcinogenic effects [16]. In particular, frozen and processed clams represent a significant source of both high-hazard polymers and microplastic intake, requiring hazard-based monitoring, intake estimations, and the inclusion of cooking-related exposure in risk assessments. Furthermore, targeted monitoring of microplastic contamination in shellfish is needed, particularly in processed products without shells, which may not be subjected to routine quality control measures, and in seafood in general, which is widely consumed globally.

The European Food Safety Authority [72] recommendations and Codex Alimentarius guidelines [73] acknowledge the risks of microplastic contamination but do not yet suggest quantitative limits for polymers in edible seafood. Nevertheless, in 2024 the European Commission [74] banned the use of Bisphenol A (BPA) for the manufacture of plastic materials intended for food contact, including polycarbonate infant feeding bottles and drinking cups, thus representing an important regulatory milestone. This measure, while not directly addressing microplastic content in food, constitutes a preliminary step toward broader regulation of plastic additives that may leach into food matrices and pose potential health risks to humans.

The high hazard indices associated with PU, PVC, and ABS found in this study highlight the necessity of reducing their environmental release by improving aquaculture, depuration, processing flows, and waste management practices and integrating microplastic monitoring in food safety. Addressing microplastic contamination through both environmental and food safety measures is crucial to reduce long-term health risks, particularly in populations with high seafood consumption, emphasising the importance of multidisciplinary approaches. Risk management strategies could also include guidelines for safe culinary practices to reduce MP exposure and consumer education campaigns focused on minimising ingestion rate.

## 5. Conclusions

Industrial processing markedly influences microplastic contamination in ready-to-cook clams, with deep-frozen de-shelled products showing higher Estimated Average Daily Intake and a considerable Polymer Hazard Index (compared with frozen clams with shells), reflecting the combined effects of shell removal, preservation liquids, and packaging materials. Fibres dominated across both product types, while polymer profiles indicate contributions from both environmental and processing-related sources, with high-hazard polymers such as PU, PVC and ABS dominating the chemical risk. These findings indicate that consumption of processed bivalves (particularly de-shelled products) represents a significant dietary route of exposure to chemically hazardous microplastics, highlighting the urgent need for integrated monitoring, standardised detection protocols, regulatory limits, and preventive strategies across harvesting, processing, and packaging, also considering cooking-related changes in microplastic bioavailability, and integrating hazard indices with dietary intake estimates to minimise potential health risks and safeguard consumer safety.

## Figures and Tables

**Table 1:** Hazard scores, classes [15] and main applications of polymers found in the samples of this study.

Polymer	Sample	Hazard Score	Hazard Class	Main Applications
**PU**	DFC-FC	13,844	5	Upholstery, sports mats, packaging bags, etc.
**PA**	DFC-FC	47	3	Bearings, automotive applications, etc.
**PS**	DFC-FC	30	2	Spectacle frames, plastic cups, packaging, etc.
**Blue paint**	FC	- *	N.c.	Boat coatings, aquaculture structure, etc.
**PVC**	DFC-FC	10,551	5	Pipes, cable insulation, garden hoses, etc.
**Nylon 6/6**	DFC-FC	63	3	Textile, automotive, industrial, consumer goods industries, etc.
**ABS**	DFC-FC	6552	5	Automotive applications, pipes, etc.
**PLC**	DFC-FC	-	N.c.	Biomedical devices, implants, scaffolds, packaging, sutures, etc.
**EPDM rubber**	FC	-	N.c.	Automotive applications, construction industry, etc.
**PVP**	FC	-	N.c.	Additive for batteries, ceramics, fibreglass, inks, inkjet paper etc.
**Polyester**	DFC-FC	1414	4	Clothing, home furnishings, etc.
**Silicon**	FC	-	N.c.	Computer chips, transistors, solar cells, sealants, lubricants, medical devices, cookware, etc.
**Cellulose acetate**	DFC-FC	-	N.c.	Textile fibres, cigarette filters, eyewear frames, etc.
**Epoxy resin**	FC	7139	5	Waterproofing, bonding, repairing ship hulls, etc.
**PP**	FC	1	1	Food packaging, microwave-proof containers, etc.
**Nylon 6**	DFC	50	2	Upholstery, carpets, ropes, gears, bearings, engine parts, etc.
**EPS**	DFC	44	3	Thermal and sound insulation, lightweight fill, protective void formers, packaging, etc.
**PVP**	DFC	-	N.c.	Batteries, ceramics, fibreglass, inks, inkjet paper, etc.
**PHB**	DFC	-	N.c.	Food packaging, medical implants, agriculture, etc.
**PE**	DFC	11	2	Toys, bottles, pipes, house ware, etc.
**POM**	DFC	1500	3-4-5	High-precision engineering, small gears, bearings, pump parts, etc.
**LDPE**	DFC	11	2	Plastic bags, food packaging, cling film, stretch and shrink films, etc.

**Legend:** * no hazard score.

**Table 2:** MP mean values and SD FC and DFC samples and of entire group samples DFC and FC.

**Frozen Clams**	**n.MP**	**MP/g**	**Deep-Frozen Clams**	**n.MP**	**MP/g**
FC 1	89.33 ± 60.28	0.67 ± 0.45	DFC 1	169 ± 152.47	2.54 ± 2.29
FC 2	55.67 ± 16.74	0.33 ± 0.1	DFC 2	252.33 ± 142.78	3.79 ± 2.14
FC 3	63.33 ± 14.04	0.37 ± 0.08	DFC 3	196.33 ± 42.1	2.95 ± 0.63
FC 4	66.68 ± 8.62	0.4 ± 0.05	DFC 4	171.67 ± 65.62	2.58 ± 0.98
FC 5	74 ± 7.55	0.44 ± 0.05	DFC 5	113.33 ± 1.53	1.36 ± 0.02
		**n.MP**		**MP/g**	
Frozen Clams		208.8 ± 38.66		0.43 ± 0.13	
Deep-Frozen Clams		541.6 ± 151		2.58 ± 0.87	

**Table 3:** Comparison between the polymeric profiles of clam samples of this study with those reported in other European and international surveys.

	PS	PVC	PU	Polyamides	PP	LDPE HDPE	Polyester	RIC 7/Others
**This study FC samples**	10.99%	7.18%	23.21%	27.88% (combined)	1.82%	–	1.99%	~25%
**This study DFC samples**	14.55%	12.74%	9.65%	28.5% (combined)	–	0.82%	0.53%	~20%
**Van Cauwenberghe & Janssen (2014) [44]**	<10%	Not reported	Not reported	10–20%	10–15%	20–30%	5–10%	Variable
**Rochman et al. (2015) [45]**	~10–20%	Not reported	Not reported	15–25%	10–20%	20–30%	5–15%	Variable
**Smith et al. (2018) [3]**	10–20%	5–15%	<5%	5–15%	15–20%	20–30%	5–10%	Variable
**Danopoulos et al. (2020) [2]**	~10–20%	~5–10%	Not reported	~10–20%	~10–15%	~20–30%	~5–10%	Variable
**Ding et al. (2021) [4]**	~5–15%	~5–10%	Not reported	10–25%	10–20%	20–35%	5–10%	Variable
**Nalbone et al. (2024) [28]**	22.22%	Not reported	Not reported	Not reported	Not reported	38.89–70.37%	Not reported	Not reported

## Data Availability

The original contributions presented in this study are included in the article. Further inquiries can be directed to the corresponding author.

## References

[1] GESAMP (2016). Sources, Fate and Effects of Microplastics in the Marine Environment: A Global Assessment.

[2] Danopoulos E., Twiddy M., Rotchell J.M., Webb S., Turner A. (2020). Microplastic contamination of bivalves: Implications for food safety and public health. Environ. Pollut..

[3] Smith M., Love D.C., Rochman C.M. (2018). Microplastics in seafood and the implications for human health. Curr. Environ. Health Rep..

[4] Ding J., Sun C., He C., Li J., Ju P., Li F. (2021). Microplastics in four bivalve species and basis for using bivalves as bioindicators of microplastic pollution. Sci. Total Environ..

[5] Giani D., Baini M., Galli M., Casini S., Fossi M.C. (2019). Microplastics occurrence in edible fish species (*Mullus barbatus* and *Merluccius merluccius*) collected in three different geographical sub-areas of the Mediterranean Sea. Mar. Pollut. Bull..

[6] Li J., Yang D., Li L., Jabeen K., Shi H. (2015). Microplastics in commercial bivalves from China. Environ. Pollut..

[7] Li J., Qu X., Su L., Zhang W., Yang D., Kolandhasamy P., Li D., Shi H. (2016). Microplastics in mussels along the coastal waters of China. Environ. Pollut..

[8] Cho Y., Shim W.J., Jang M., Han G.M., Hong S.H. (2019). Abundance and characteristics of microplastics in market bivalves from South Korea. Environ. Pollut..

[9] Nalbone L., Cincotta F., Giarratana F., Ziino G., Panebianco A. (2021). Microplastics in fresh and processed mussels sampled from fish shops and large retail chains in Italy. Food Control.

[10] Dambrosio A., Cometa S., Capuozzo F., Ceci E., Derosa M., Quaglia N.C. (2023). Occurrence and characterization of microplastics in commercial mussels (*Mytilus galloprovincialis*) from Apulia region (Italy). Foods.

[11] Quaglia N.C., Capuozzo F., Ceci E., Cometa S., Di Pinto A., Mottola A., Piredda R., Dambrosio A. (2023). Preliminary survey on the occurrence of microplastics in bivalve mollusks marketed in Apulian fish markets. Ital. J. Food Saf..

[12] Nakazawa N., Okazaki E. (2020). Recent research on factors influencing the quality of frozen seafood. Fish. Sci..

[13] (2022). Standard Practice for Coding Plastic Manufactured Articles for Resin Identification.

[14] Hidalgo-Ruz V., Gutow L., Thompson R.C., Thiel M. (2012). Microplastics in the marine environment: A review of the methods used for identification and quantification. Environ. Sci. Technol..

[15] Xu P., Peng G., Su L., Gao Y., Gao L., Li D. (2018). Microplastic risk assessment in surface waters: A case study in the Changjiang Estuary, China. Mar. Pollut. Bull..

[16] Lithner D., Larsson Å., Dave G. (2011). Environmental and health hazard ranking and assessment of plastic polymers based on chemical composition. Sci. Total Environ..

[17] Istituto Nazionale di Statistica (ISTAT) (2022). Consumi Alimentari Delle Famiglie Italiane.

[18] Li J., Zhang L., Dang X., Su L., Jabeen K., Wang H., Wang Z. (2022). Effects of cooking methods on microplastics in dried shellfish. Sci. Total. Environ..

[19] Zhu L., Li X., Wang X. (2022). Microplastics in commercial clams from the intertidal zone of the South Yellow Sea, China: Distribution, migration, and mitigation strategies. Front. Mar. Sci..

[20] Hahladakis J.N., Velis C.A., Weber R., Iacovidou E., Purnell P. (2018). An overview of chemical additives present in plastics: Migration, release, fate and environmental impact during their use, disposal and recycling. J. Hazard. Mater..

[21] Sharma P. (2024). Microplastic contamination in food processing: Role of packaging materials. Food Sci. Eng..

[22] Wright S.L., Kelly F.J. (2017). Plastic and human health: A micro issue?. Environ. Sci. Technol..

[23] Pfohl P., Wagner M., Meyer L., Domercq P., Praetorius A., Hüffer T., Hofmann T., Wohlleben W. (2022). Environmental degradation of microplastics: How to measure fragmentation rates to secondary micro- and nanoplastic fragments and dissociation into dissolved organics. Environ. Sci. Technol..

[24] Hussain K.A., Romanova S., Okur I., Zhang D., Kuebler J., Huang X., Wang B., Fernandez-Ballester L., Lu Y., Schubert M. (2023). Assessing the release of microplastics and nanoplastics from plastic containers and reusable food pouches: Implications for human health. Environ. Sci. Technol..

[25] Chinglenthoiba C., Lani M.N., Anuar S.T., Amesho K.T., KL P., Santos J.H. (2025). Microplastics in food packaging: Analytical methods, health risks, and sustainable alternatives. J. Hazard. Mater. Adv..

[26] Kwon J.H., Kim J.W., Pham T.D., Tarafdar A., Hong S., Chun S.H., Lee S., Kang D., Kim J., Kim S. (2020). Microplastics in food: A review on analytical methods and challenges. Int. J. Environ. Res. Public Health.

[27] Avio C.G., Gorbi S., Milan M., Benedetti M., Fattorini D., d’Errico G., Pauletto M., Bargelloni L., Regoli F. (2015). Pollutants bioavailability and toxicological risk from microplastics to marine mussels. Environ. Pollut..

[28] Nalbone L., Giarratana F., Genovese M., Panebianco A. (2024). Occurrence of microplastics in store-bought fresh and processed clams in Italy. Mar. Pollut. Bull..

[29] Xu Z., Huang L., Xu P., Lim L., Cheong K.-L., Wang Y., Tan K. (2024). Microplastic pollution in commercially important edible marine bivalves: A comprehensive review. Food Chem. X.

[30] Kuttykattil A., Suresh S., Rajan R. (2023). Consuming microplastics? Investigation of commercial salt contamination. Environ. Sci. Pollut. Res..

[31] Rubio-Armendáriz C., Alejandro-Vega S., Paz-Montelongo S., Gutiérrez-Fernández Á.J., Carrascosa-Iruzubieta C.J., Hardisson-de la Torre A. (2022). Microplastics as emerging food contaminants: A challenge for food safety. Int. J. Environ. Res. Public Health.

[32] Prata J.C., da Costa J.P., Lopes I., Duarte A.C., Rocha-Santos T. (2020). Environmental exposure to microplastics: An overview on possible human health effects. Sci. Total. Environ..

[33] Cai Y., Mitrano D.M., Hufenus R., Nowack B. (2021). Formation of fiber fragments during abrasion of polyester textiles. Environ. Sci. Technol..

[34] Browne M.A., Crump P., Niven S.J., Teuten E., Tonkin A., Galloway T., Thompson R. (2011). Accumulation of microplastic on shorelines worldwide: Sources and sinks. Environ. Sci. Technol..

[35] De Falco F., Di Pace E., Cocca M., Avella M. (2019). The contribution of washing processes of synthetic clothes to microplastic pollution. Sci. Rep..

[36] Galloway T.S., Cole M., Lewis C. (2017). Interactions of microplastic debris throughout the marine ecosystem. Nat. Ecol. Evol..

[37] Cox K.D., Covernton G.A., Davies H.L., Dower J.F., Juanes F., Dudas S.E. (2019). Human consumption of microplastics. Environ. Sci. Technol..

[38] Toussaint B., Raffael B., Angers-Loustau A., Gilliland D., Kestens V., Petrillo M., Rio-Echevarria I.M., Van den Eede G. (2019). Review of micro- and nanoplastic contamination in the food chain. Food Addit. Contam. Part A.

[39] Hoang H.G., Nguyen N.S.H., Zhang T., Tran H.-T., Mukherjee S., Naidu R. (2025). A review of microplastic pollution and human health risk assessment: Current knowledge and future outlook. Front. Environ. Sci..

[40] De Guzman M.K., Andjelković M., Jovanović V., Jung J., Kim J., Dailey L.A., Rajković A., De Meulenaer B., Ćirković Veličković T. (2022). Comparative profiling and exposure assessment of microplastics in differently sized Manila clams from South Korea by μFTIR and Nile Red staining. Mar. Pollut. Bull..

[41] Li Y., Tao L., Wang Q., Wang F., Li G., Song M. (2023). Potential health impact of microplastics: A review of environmental distribution, human exposure, and toxic effects. Environ. Health.

[42] Hassan M.M., Nohor N. (2025). Bridging the microplastics–public health research gap: A call for translational action in vulnerable populations. Health Sci. Rep..

[43] Vitale D., Spinelli A., Picó Y. (2023). Microplastics detected in sediments and rock substrate of marine areas with ghost nets. J. Mar. Sci. Eng..

[44] Van Cauwenberghe L., Janssen C.R. (2014). Microplastics in bivalves cultured for human consumption. Environ. Pollut..

[45] Rochman C.M., Tahir A., Williams S.L., Baxa D.V., Lam R., Miller J.T., Teh F.-C., Werorilangi S., Teh S.J. (2015). Anthropogenic debris in seafood: Plastic debris and fibers from textiles in fish and bivalves sold for human consumption. Sci. Rep..

[46] Fred-Ahmadu O.H., Ahmadu F.O., Adedapo A.E., Oghenovo I., Ogunmodede O.T., Benson N.U. (2024). Microplastics and chemical contamination in aquaculture ecosystems: The role of climate change and implications for food safety—A review. Environ. Sci. Eur..

[47] Mutić T., Mutić J., Ilić M., Jovanović V., Aćimović J., Andjelković B., Stanić-Vucinić D., de Guzman M.K., Andjelkovic M., Turkalj M. (2024). The Global Spread of Microplastics: Contamination in Mussels, Clams, and Crustaceans from World Markets. Foods.

[48] Digka N., Patsiou D., Hatzonikolakis Y., Raitsos D.E., Skia G., Koutsoubas D., Dimitriadis C., Tsangaris C. (2024). Microplastic ingestion in mussels from the East Mediterranean Sea: Exploring its impacts in nature and controlled conditions. Sci. Total. Environ..

[49] Abbasi F., De-la-Torre G.E., Renner G., Green D.S., Schmidt T.C., Dobaradaran S. (2025). A review of occurrence and concentrations of cellulose acetate and other artificial cellulose microfibers in aquatic environmental matrices: An indicator of cigarette butts’ contamination?. J. Hazard. Mater. Adv..

[50] Acharjee S.A., Bharali P., Gogoi B., Sorhie V., Walling B., Alemtoshi (2023). PHA-based bioplastic: A potential alternative to address microplastic pollution. Water Air Soil Pollut..

[51] Yang S., Gu C., Jiao Y., Yang Q. (2023). Research on the presence of cigarette butts and their leaching of chemical pollutants and microparticles: The case of Dalian, China. Front. Mar. Sci..

[52] Stoica M., Bichescu C.I., Crețu C.-M., Dragomir M., Ivan A.S., Podaru G.M., Stoica D., Stuparu-Crețu M. (2024). Review of bio-based biodegradable polymers: Smart solutions for sustainable food packaging. Foods.

[53] Robison-Smith C., Cable J. (2024). Invisible plastics problem in intensive aquaculture: The case of polyvinylpyrrolidone. Rev. Aquac..

[54] Visciano P. (2024). Environmental contaminants in fish products: Food safety issues and remediation strategies. Foods.

[55] Giri S., Lamichhane G., Khadka D., Devkota H.P. (2024). Microplastics contamination in food products: Occurrence, analytical techniques and potential impacts on human health. Curr. Res. Biotechnol..

[56] Nam S.H., Seo Y.M., Kim M.G. (2010). Bisphenol A migration from polycarbonate baby bottle with repeated use. Chemosphere.

[57] Qin J., Liang B., Peng Z., Lin C. (2021). Generation of microplastic particles during degradation of polycarbonate films in various aqueous media and their characterization. J. Hazard. Mater..

[58] Athanasopoulou E., Power D.M., Flemetakis E., Tsironi T. (2025). Towards the Rational Use of Plastic Packaging to Reduce Microplastic Pollution: A Mini Review. J. Mar. Sci. Eng..

[59] Baechler B.R., Granek E.F., Hunter M.V., Conn K.E. (2020). Microplastic concentrations in two Oregon bivalve species: Spatial, temporal, and species variability. Limnol. Oceanogr. Lett..

[60] Alak G., Köktürk M., Atamanalp M. (2024). Evaluation of phthalate migration potential in vacuum-packed. Sci. Rep..

[61] Gupta R.K., Pipliya S., Karunanithi S., Eswaran U G.M., Kumar S., Mandliya S., Srivastav P.P., Suthar T., Shaikh A.M., Harsányi E. (2024). Migration of chemical compounds from packaging materials into packaged foods: Interaction, mechanism, assessment, and regulations. Foods.

[62] Robertson G.L. (2005). Food Packaging: Principles and Practice.

[63] Alamri M.S., Qasem A.A.A., Mohamed A.A., Hussain S., Ibraheem M.A., Shamlan G., Alqah H.A., Qasha A.S. (2021). Food packaging’s materials: A food safety perspective. Saudi J. Biol. Sci..

[64] European Food Safety Authority (2021). EFSA Scientific Colloquium 25—A coordinated approach to assess the human health risks of micro- and nanoplastics in food. EFSA Support. Publ..

[65] Siddique M.A.M., Das K., Shazada N.E., Walker T.R. (2025). Microplastic ingestion and potential risk assessment on commercial and non-commercial marine fish in the Bay of Bengal. Water Air Soil Pollut..

[66] Simon-Sánchez L., Vianello A., Kirstein I.V., Molazadeh M.-S., Lorenz C., Vollertsen J. (2024). Assessment of microplastic pollution and polymer risk in the sediment compartment of the Limfjord, Denmark. Sci. Total. Environ..

[67] Papini G., Rakaj A. (2025). Microplastic retention in European flat oyster Ostrea edulis cultured in two Mediterranean basins. npj Emerg. Contam..

[68] Turner A., Filella M. (2021). Polyvinyl chloride in consumer and environmental plastics, with a particular focus on metal-based additives. Environ. Sci. Process. Impacts.

[69] Atkinson S.A., Caballero B. (2013). Nutritional requirements of infants. Encyclopedia of Human Nutrition.

[70] (2023). EUMOFA. https://eumofa.eu/household-consumption-of-fresh-products.

[71] Schwabl P., Köppel S., Königshofer P., Bucsics T., Trauner M., Reiberger T., Liebmann B. (2019). Detection of various microplastics in human stool: A prospective case series. Ann. Intern. Med..

[72] European Food Safety Authority (EFSA) (2016). Presence of microplastics and nanoplastics in food, with particular focus on seafood. EFSA J..

[73] (2020). General Standard for Contaminants and Toxins in Food and Feed.

[74] European Commission (2024). Commission Regulation (EU) 2024/3190 of 19 December 2024 on the Use of Bisphenol A (BPA) and Other Bisphenols and Bisphenol Derivatives with Harmonised Classification for Specific Hazardous Properties in Certain Materials and Articles Intended to Come into Contact with Food, Amending Regulation (EU) No 10/2011 and Repealing Regulation (EU) 2018/213.

